# Calcium/calmodulin-dependent kinase kinase 2 regulates hematopoietic stem and progenitor cell regeneration

**DOI:** 10.1038/cddis.2017.474

**Published:** 2017-10-05

**Authors:** Luigi Racioppi, William Lento, Wei Huang, Stephanie Arvai, Phuong L Doan, Jeffrey R Harris, Fernando Marcon, Helder I Nakaya, Yaping Liu, Nelson Chao

**Affiliations:** 1 Division of Hematological Malignancies and Cellular Therapy, Department of Medicine, Duke University Medical Center, Durham, NC, USA; 2 Department of Molecular Medicine and Medical Biotechnology, University of Naples Federico II, Naples, Italy; 3 Department of Pathophysiology and Toxicology, School of Pharmaceutical Sciences, University of São Paulo, São Paulo, Brazil

## Abstract

Hematopoietic stem and progenitor cells (HSPCs) are predominantly quiescent in adults, but proliferate in response to bone marrow (BM) injury. Here, we show that deletion of Ca^2+^/calmodulin (CaM)-dependent protein kinase kinase 2 (CaMKK2) promotes HSPC regeneration and hematopoietic recovery following radiation injury. Using Camkk2-enhanced green fluorescent protein (EGFP) reporter mice, we found that Camkk2 expression is developmentally regulated in HSPC. Deletion of Camkk2 in HSPC results in a significant downregulation of genes affiliated with the quiescent signature. Accordingly, HSPC from Camkk2 null mice have a high proliferative capability when stimulated *in vitro* in the presence of BM-derived endothelial cells. In addition, *Camkk2* null mice are more resistant to radiation injury and show accelerated hematopoietic recovery, enhanced HSPC regeneration and ultimately a prolonged survival following sublethal or lethal total body irradiation. Mechanistically, we propose that CaMKK2 regulates the HSPC response to hematopoietic damage by coupling radiation signaling to activation of the anti-proliferative AMP-activated protein kinase. Finally, we demonstrated that systemic administration of the small molecule CaMKK2 inhibitor, STO-609, to irradiated mice enhanced HSPC recovery and improved survival. These findings identify CaMKK2 as an important regulator of HSPC regeneration and demonstrate CaMKK2 inhibition is a novel approach to promoting hematopoietic recovery after BM injury.

Hematopoietic stem and progenitor cells (HSPCs) reside in specialized bone marrow (BM) niches that provide signals to ensure blood production and maintain the long-term hematopoietic stem cell (LT-HSC) pool. Extensive studies of the niche have identified several cell types such as osteoblasts,^1^ endothelial cells,^2^ osteomacs,^3^ regulatory T cells^4^ and sympathetic neurons^5^ as contributors of the physiologic microenvironment.^6, 7^ These cells engage HSPC through both physical contacts and soluble paracrine signaling molecules including CXC chemokine ligand 12 (CXCL12), stem cell factor (SCF), non-canonical and canonical Wnt ligands, and epidermal growth factor^8, 9^ to control niche retention and self-renewal. Although these molecules may trigger calcium transients, the role of calcium-dependent cascades in the mechanism regulating HSCP regeneration has not been elucidated.^10, 11, 12, 13^

Calmodulin (CaM) is the primary intercellular calcium sensor and binding to free cytosolic Ca^2+^ causes conformational changes that facilitate its interaction with the multifunctional Ser/Thr kinases Ca^2+^/CaM-dependent protein kinase I, IV (CaMKI and CaMKIV, respectively) and Ca^2+^/CaM-dependent protein kinase kinase 1 (CaMKK1) and CaMKK2 to activate Ca^2+^/CaM-dependent signaling cascades.^14, 15^ CaMKK2 activation permits phosphorylation of CaMKI, CaMKIV and the adenosine monophosphate activated protein kinase (AMPK).^16^ The expression of CaMKK2 is relatively cell type restricted and outside the brain it is found in osteoblasts,^17^ macrophages^18^ and myeloid progenitors.^19^

Here, we demonstrate CaMKK2 functions as a critical kinase that regulates the regeneration of HSPC. CaMKK2 deficiency downregulates genes affiliated with stem cell quiescence and causes a HSPC hyper-proliferative phenotype *in vitro* and accelerates hematopoietic recovery following radiation injury *in vivo*. Mechanistically, we demonstrate CaMKK2 is required to link radiation injury with AMPK activation and p53 accumulation. Importantly, the transient inhibition of CaMKK2 with the small molecule CaMKK2 kinase inhibitor STO-609^20^ improves survival and hematopoietic regeneration.

## Results

### CaMKK2 expression is enriched in HSPC *in vivo*

**Figure 1:**
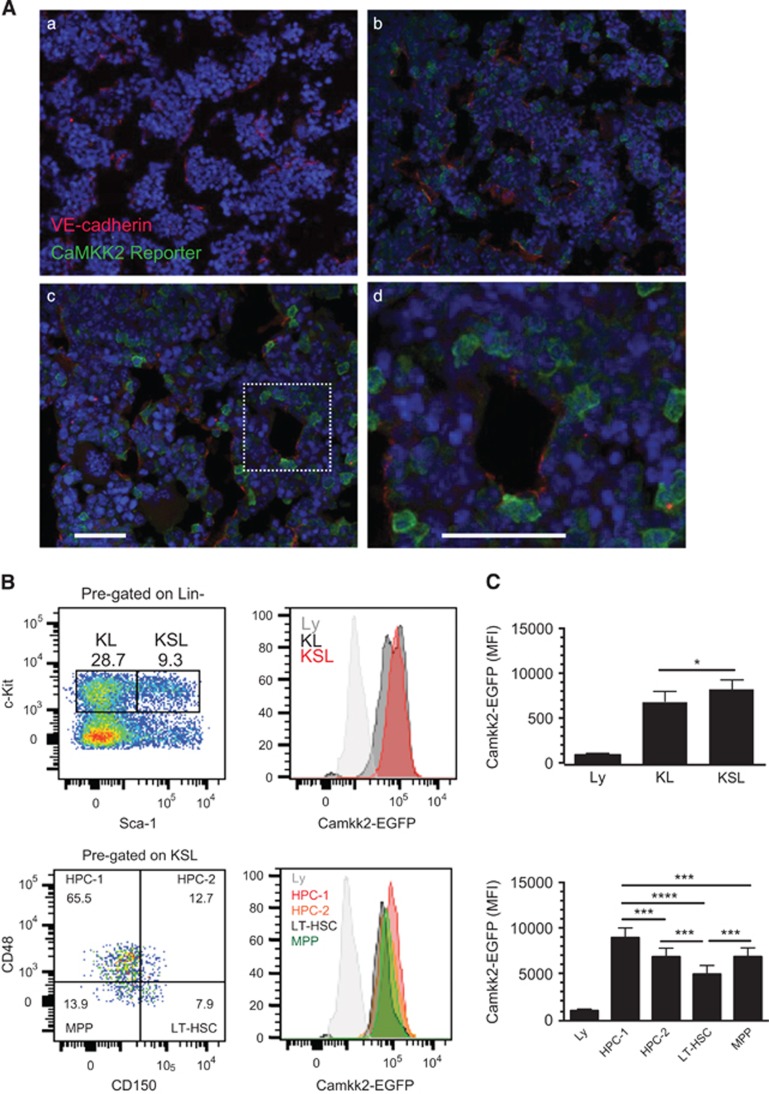
Camkk2 expression is enriched in primitive HSPCs *in vivo.* (**A**) Femurs were harvested from control and Camkk2-EGFP reporter mice and sectioned for immunofluorescent staining with VE-cadherin and anti-GFP antibodies (**A**a, **A**b; low magnification, **A**c and insets high magnification) (*n*=3 per genotype). (**B**) BM cells from control and Camkk2-EGFP reporter mice were isolated, stained to identify HSPC subsets and analyzed by flow cytometry (top and bottom panels). (**C**) The reporter expression is shown relative to lymphocyte EGFP intensity, which is considered CaMKK2 negative. The relative EGFP expression is quantitated in the right panels (*n*=6 mice/genotype). Bars graph reports mean±S.E.M. *, *** and **** refer to *P*-values < 0.05, 0.005 and 0.001, respectively

We used CaMKK2-enhanced green fluorescent protein (EGFP) mice to determine the location and phenotype of Camkk2-expressing cells within the BM microenvironment. The analysis of reporter bone sections by immunofluorescence revealed activity in individual single cells throughout the BM (Figure 1A). A subset of EGFP-positive cells was located adjacent to the vascular endothelial marker VE-cadherin (Figures 1Ac–d, high-magnification inset). We found that approximately 20% of lineage (Lin)+ cells were EGFP positive by flow cytometry. The reporter was active in 25–40% of Lin^-^cKit^-^ (CD117) stem cell antigen-1- (Sca^-^) cells and 5–10% of the Lin^-^cKit^-^Sca^+^ cells. Conversely, the EGFP reporter was detectable in 95–100% of Lin^-^cKit^+^ cells (Figures 1B and C). A detailed analysis of the HSPC compartment revealed robust reporter activation in Lin^-^cKit^+^Sca^-^ (KL) and Lin^-^cKit^+^Sca^+^ (KSL) cells (Figures 1B and C, top panels). Additional KSL subset analyses using the signaling lymphocyte activation molecules (SLAM) markers CD150 and CD48 showed EGFP expression in the LT-HSC population defined as KSL CD150^+^CD48^-^ and multipotent progenitors (MPP, KSL CD150^-^CD48^-^; Figures 1B and C, bottom panels). The reporter was highly active in committed hematopoietic progenitor populations 1 and 2 (HPC-1 and HPC-2) defined as KSL CD150^-^CD48^+^ and CD150^+^CD48^+^^21^ (Figures 1B and C, bottom panels). Cumulatively, these data suggest the capacity to initiate Ca^2+^-dependent signaling via CaMKK2 is enriched in the most primitive hematopoietic stem cells.

### Camkk2 regulates the HSPC transcriptional program

**Figure 2:**
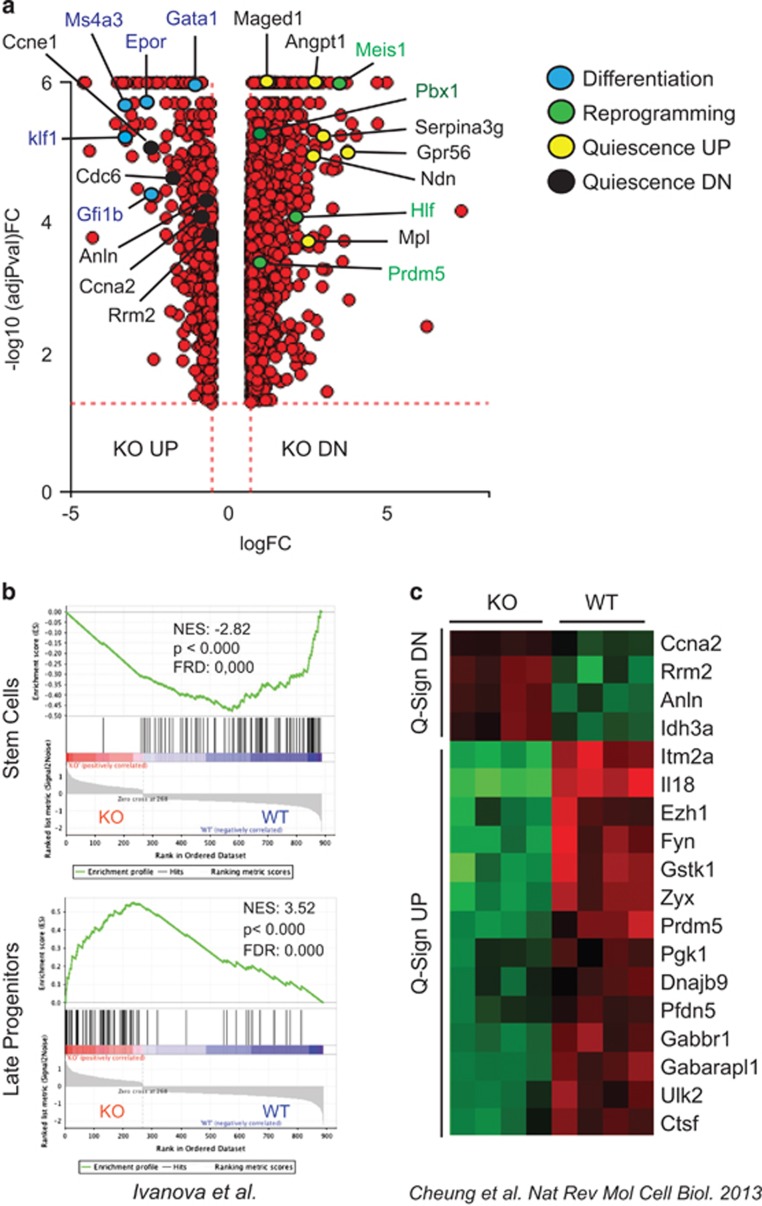
Camkk2 regulates transcriptional program of hematopoietic stem cells. (**a**) Volcano plot comparison of DEGs in KSL cells isolated from BM of Camkk2 null and control mice (WT and KO, respectively). Genes downregulated or upregulated in KO compared with WT are indicated as KO DN and KO UP, respectively. Color dots indicate genes involved in the regulation of differentiation or reprogramming of mature hematopoietic cells to HSC. (**b**) GSEA of microarray data shows that gene signatures for genes enriched in hematopoietic stem cells are significantly downregulated in KO KSL (upper). In contrast, genes enriched in late progenitors are significantly upregulated in KO KSL (lower). (**c**) Loss of Camkk2 downregulates the quiescent gene signature in stem cells. Heatmap represents DEGs in quiescent stem cell signature. Q-Sign DN and UP indicate genes downregulated and upregulated in quiescent stem cells. The color key of heatmaps indicates row-wise scaled RPKM values (z-score)

To identify gene sets controlled by Camkk2 in HSPC, we performed microarray analyses on KSL cells isolated from WT and Camkk2 null mice and found 1289 genes were significantly downregulated and 533 genes were upregulated in Camkk2 null KSL compared with WT (Figure 2a). The lists of the top differentially expressed genes (DEGs) are reported in Supplementary Table S1 and S2. The gene set enrichment analysis (GSEA)^22^ showed genes downregulated in Camkk2 null KSL were enriched in HSC,^23, 24^ early progenitors,^24^ and lymphoid-myeloid-affiliated genes (s-myly) primed in HSC^24^ (Figure 2b, upper panel; Supplementary Figure S1A). The HSC-affiliated downregulated genes in Camkk2 KSL included Hlf, Meis1, Pbx-1 and Prdm5. These genes represent four of the eight genes capable of reprogramming committed murine blood cells into HSC (Figure 2a).^25^ Conversely, genes affiliated with the late progenitor signature^23^ (Figure 2b, lower panel), erythroid genes primed in HSC,^24^ and granulocyte macrophage progenitor (GMP)-specific genes (d-my)^24^ were significantly upregulated in Camkk2 null KSL (Supplementary Figures S1B and C).

The pathways over represented by Camkk2 null KSL DEGs included cell adhesion molecules (CAMs, *P*=8.218e-8) and chemokine signal pathway (*P*=0.0275; Supplementary Table S2). We analyzed the gene expression affiliated with the quiescent signature in various stem cells including hematopoietic, muscle and hair follicle stem cells.^26^ We found 25% (18 of 71) of genes affiliated with a quiescent stem cell signature (Q-Sign) were differentially expressed in Camkk2 null KSL. Interestingly, 14 of 49 genes upregulated in Q-Sign (Q-Sign UP) were downregulated in Camkk2 KO KSL (Figure 2c). Moreover, none of the Q-Sign UP genes were upregulated in Camkk2 null KSL (Figure 2c; *P*=0.0144). None of the 22 genes downregulated in quiescent stem cells (Q-Sign DN) were downregulated in Camkk2 null KSL. In contrast, four Q-Sign DN genes (Ccna2, Rrm2, Anln and Idh3) were upregulated in Camkk2 null compared with WT KSL (Figure 2c; *P*-value=0.0075). This finding was corroborated using gene signatures specifically affiliated with HSC quiescence and proliferative status (HQ-sign and HP-sign, respectively).^27^ We found 15% of HQ-sign genes (40 of 264) were significantly downregulated in Camkk2 null KSL. However, only 3 of 264 genes were significantly upregulated in Camkk2 null KSL (Supplementary Figures S2A–C; *P*=0.0001). The data indicate 12 of 313 genes in the HP-Sign were upregulated in Camkk2 null KSL compared with WT and 3 of 264 were downregulated in Camkk2 null KSL (Supplementary Figure S2A and B; *P*=0.0632). Collectively, these findings indicate the deletion of Camkk2 downregulates genes controlling HSPC quiescence and concurrently upregulates genes affiliated with the late progenitor signature.

### Camkk2 null HSPC have increased proliferation *in vitro*

**Figure 3:**
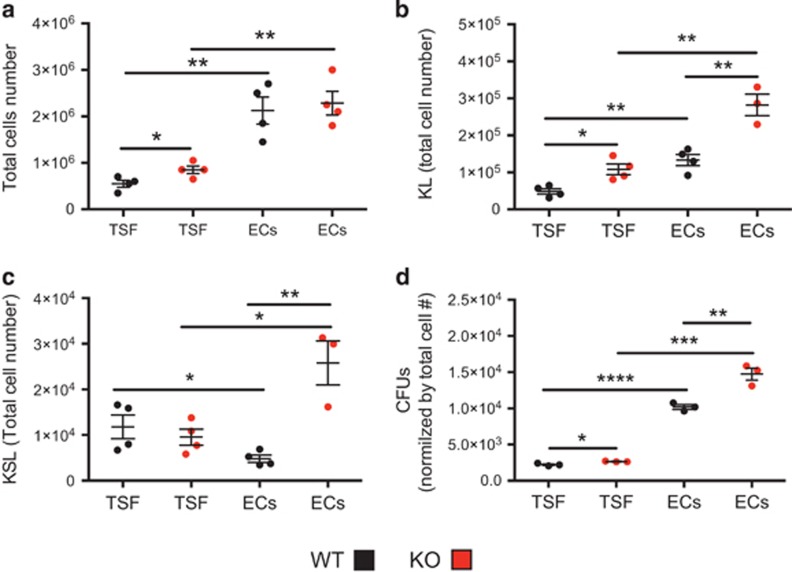
Camkk2 null hematopoietic stem cells have increased proliferation *in vitro.* KSL cells were sorted from WT and Camkk2 null mice and cultured with TPO, SCF and Flt-3L in the presence or absence of BM endothelial cells (TSF and ECs, respectively). Cell were harvested and analyzed on day 7. (**a**) Total cells number. (**b** and **c**) Absolute numbers of KL and KSL cells. (**d**) Cells recovered at day 7 were plated in methylcellulose media for colony formation and colonies (CFUs). Graphs report total CFUs normalized by total cell expansion. The experiment was replicated twice. Bars graph reports mean±S.E.M. *P<0.05, **P<0.01, ***P<0.005. **** *P*<0.001

We cultured KSL cells sorted from control and Camkk2 null mice with thrombopoietin, SCF and Flt-3-ligand cytokines (TSF) to analyze the effect of Camkk2 ablation on HSPC proliferation in response to cytokines. In addition, we mimicked the vascular niche microenvironment using non-contact cultures with primary BM-derived endothelial cells (BMECs). After 7 days, the numbers of total KL and KSL cells were assessed. The cells recovered from cultures were then functionally analyzed in methylcellulose cultures to examine colony-forming unit (CFU) capacity. Regardless of the genotype, significantly more total cells were recovered from co-culture with BMEC compared with TSF alone (Figure 3a). There was a slight increase in the numbers of total and KL cells found in TSF cultures of Camkk2 null KSL compared with WT cells (Figures 3b and c). Interestingly, the presence of BMEC resulted in a more robust increase in both KL and KSL recovered from Camkk2 null KSL compared with WT cultures (Figures 3b and c). The progeny derived from Camkk2 null KSL cultured in the presence of TSF had a modest increase in functional colony formation compared with WT cells (Figure 3d). In contrast, progeny derived from Camkk2 null KSL cultured in the presence of BMEC generated a significantly higher number of CFUs compared with WT (Figure 3d). Cumulatively, these results show Camkk2 null KSL cells have significantly higher proliferative ability than WT cells in culture conditions mimicking the hematopoietic niche.

### Camkk2 null mice have improved survival and accelerated hematopoietic recovery following total body irradiation (TBI)

**Figure 4:**
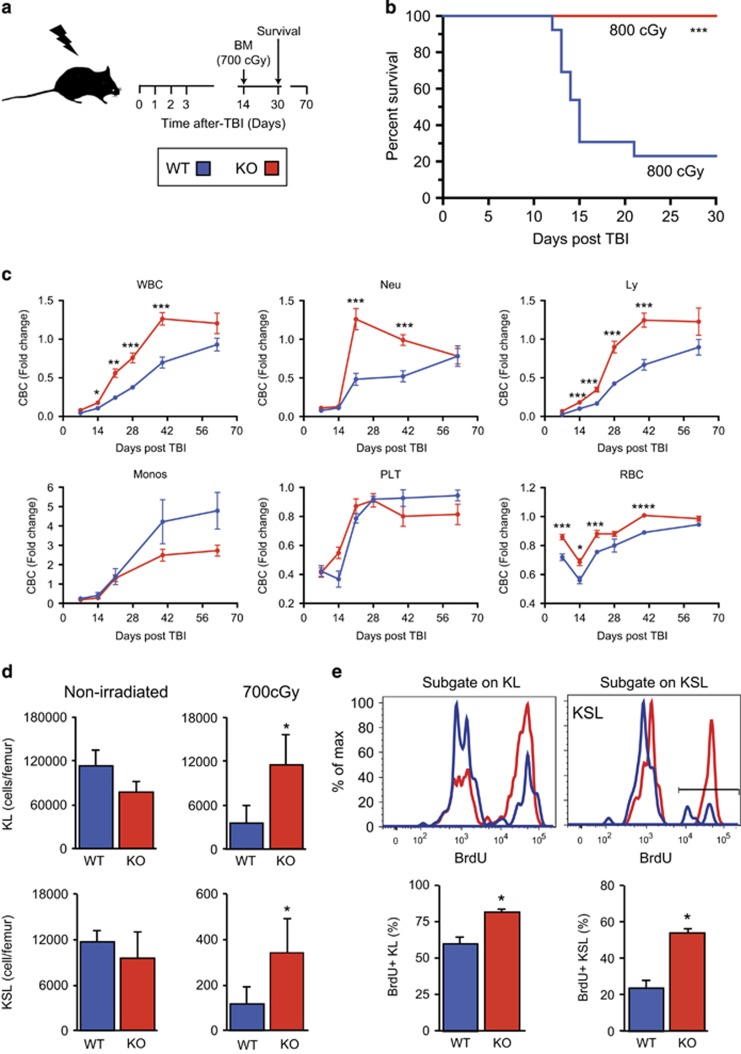
Camkk2 null mice have improved survival and accelerated hematopoietic recovery following TBI. (**a**) Scheme of TBI. Mice were TBI and monitored for survival, blood cell count (CBC) and BM recovery. (**b**) Survival of WT and Camkk2 null mice (WT and KO, respectively) irradiated with 800cGy (*n*=14 mice per genotype). The blue lines indicate control and the red lines indicate Camkk2 null mice. (**c**) Hematopoietic recovery in WT and KO mice sublethally irradiated with 700cGy TBI and bled for CBC analysis of total WBCs, platelets (PLT), RBCs, neutrophils (NE), monocytes (Mo) and lymphocytes (Ly) (*n*=6 and 9 mice for WT and Camkk2 null mice, respectively). (**d**) WT and KO mice (*n*=10 per group) were irradiated with 700cGy TBI and euthanized 14 days after irradiation. WT and KO non-irradiated mice were used as controls (*n*=6 mice per group). Upper and lower bar graphs report mean ±S.E.M. of KL and KSL, respectively. (**e**) BrdU incorporation in KL and KSL cells *in vivo* during regeneration. WT and KO mice were irradiated with 700cGy TBI, and after 14 days were pulsed with BrdU *in vivo* for 2 h before killing. Dot plots of KL and KSL cells and BrdU incorporation on day 14 after radiation (top panels). BrdU staining FACS profiles in KL and KSL subsets (upper panels). Bars graph reports mean±S.E.M. The percentage of BrdU^+^ cells is shown in lower graphs (bottom panels; *n*=6 per genotype). *P<0.05, **P<0.01, ***P<0.005, ****P<0.001

We previously reported that under homeostatic conditions, Camkk2 ablation does not affect survival and proliferation of the KSL population *in vivo.*^19^ However, we observed Camkk2 null HSPC have an increased ability to proliferate *in vitro* (Figure 3). Therefore, we hypothesized CaMKK2 restrains the proliferation of HSPC. Thus, Camkk2 ablation would accelerate the hematological recovery following BM damage. To test this hypothesis, we injured the hematopoietic compartment *in vivo* using total body *γ*-irradiation (TBI; 700-900cGy) and then monitored mice for survival, blood counts, and BM recovery (Figure 4a).

Using a single dose of 800cGy TBI, which is sufficient to kill approximately 80% of control animals in 30 days (LD_80/30_), we found 100% of Camkk2 null animals and 20% of control animals survived for >30 days (Figure 4b, *P*<0.0001). Although a higher radiation dose (900cGy, LD_100/15;_ Supplementary Figure S3A) killed all control animals, approximately 40% of Camkk2 null animals survived for >30 days (Figure 3a, *P*<0.01).

We then monitored the peripheral blood cell count (CBC) recovery of irradiated mice using a single sublethal radiation dose (700cGy). Non-irradiated Camkk2 null mice have significantly more monocytes and less white blood cells (WBCs), neutrophils, and lymphocytes in blood compared with WT mice. The number of red blood cells (RBCs) and platelets were comparable in WT and Camkk2 null mice (Supplementary Figure S3). The irradiated Camkk2 null mice have a better ability to regenerate neutrophils, leukocytes and RBC compared with WT mice (Supplementary Figures S3B and S4C). In addition, the number of monocytes was also significantly higher in TBI Camkk2 null mice compared with WT mice (Supplementary Figure S3B). However, when normalized for the basal level, we found WT and Camkk2 null mice showed a comparable ability to regenerate monocytes and platelets following TBI (Figure 4c and Supplementary Figure S3B).

To examine the BM content during regeneration, we killed 700cGy TBI control and Camkk2 null animals on day 14 and isolated BM for histology and flow cytometry analyses. The histological analysis indicated Camkk2 null animals had qualitatively higher BM cellularity than control mice (Supplementary Figure S4A, left) and there were significantly more cells in the marrow of irradiated Camkk2 null animals compared with WT mice (Supplementary Figure S4B; *P*<0.05). There were comparable numbers of WBMC in non-irradiated WT and Camkk2 null mice (Supplementary Figure 4B).

We assessed whether the accelerated hematopoietic recovery was mediated by enhanced HSPC regeneration by analyzing the KL and KSL cell content in BM on day 14 following 700cGy TBI. Although we found a trend of reduced absolute KL and KSL number in non-irradiated marrow, there were significantly more KL and KSL cells during hematopoietic regeneration (Figure 4d). The SLAM KSL subsets were analyzed in TBI and non-irradiated mice based on the expression of CD48 and CD150 (Supplementary Figure S4C). The data show an increase in the percentage of LT-HSC in Camkk2 null mice under both homeostatic conditions and following TBI (Supplementary Figure S4D, left). In addition, the percentage of HPC-2 subsets was higher only in TBI Camkk2 null mice compared with control mice (Supplementary Figure S4D, right). Cumulatively, these data indicate Camkk2 null mice have accelerated HSPC recovery following radiation injury and this accounted for the faster hematopoietic recovery.

### Total body irradiated Camkk2 null mice have more proliferating HSPC in BM

We examined the *in vivo* sensitivity of HSPC to radiation-induced apoptosis to determine the cellular basis of the accelerated hematopoietic recovery in Camkk2 null mice. As previously reported,^19^ Camkk2 deficiency does not impair the survival of HSPC in non-irradiated mice (Supplementary Figures S5A and S5B). Our data show a slight increase in live (10–15%) Camkk2 null KSL cells compared with WT KSL cells, but no differences in KL cells were observed 24 h after 450cGy TBI (Supplementary Figures S5C and S5D).

We examined the proliferative response of the KL and KSL cells in the BM of regenerating control and Camkk2 null mice on day 14 after 700cGy TBI. We found significantly more 5-bromo-2'-deoxyuridine (BrdU) incorporation in Camkk2 null KL and KSL cells during this period (Figure 4e). The results indicate approximately 20% of control KSL cells are BrdU+, but >50% of Camkk2 null KSL cells are in cycle at day 14. To test whether the functional capacity of regenerated stem cells was decreased by the enhanced proliferation, we performed competitive transplantation assays using control and Camkk2 null KSL CD34^-^ cells harvested from the BM of 200cGy TBI mice. We found radiation did not induce differential HSC exhaustion or cause significant lineage skewing in the peripheral blood of recipient mice (Supplementary Figure S6). Cumulatively, these data demonstrate the loss of Camkk2 enhanced the proliferation of regenerating HSPC, which accelerates hematopoietic recovery.

### Camkk2 null HSPC have a cell-intrinsic enhanced regenerative capability *in vivo*

**Figure 5:**
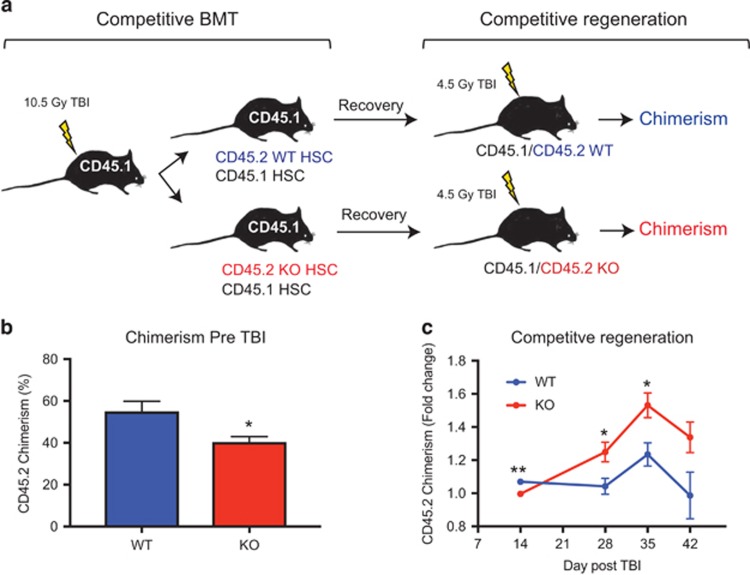
Camkk2 null HSC have a cell-intrinsic enhanced regenerative capability *in vivo*. KSL CD34^-^ cells were isolated from WT and Camkk2 null mice (WT and KO, respectively) and transplanted in lethally irradiated recipient mice with CD45.1 competitor BM. The recipient mice receiving WT or KO KSL CD34^-^ cells were monitored for 4 months. Subsequently, mice showing comparable percentages of WT or KO donor CD45.2 cells were irradiated with 450cGy TBI and bled weekly after irradiation (*n*= 6 per group). (**a**) Scheme of the experiment. (**b**) CD45.2 chimerism in mice reconstituted with WT or KO KSL CD34^-^ before receiving 4.5Gy TBI. (**c**) Donor CD45.2 chimerism was monitored by flow cytometry and the results are expressed as fold change over the basal level (pre-TBI). Bars graph reports mean±S.E.M. *P<0.05, **P<0.01

To establish the cell-autonomous function of CaMMK2 on the regenerative capability of HSPC *in vivo,* we isolated KSL CD34^-^ cells from WT and Camkk2 null mice (CD45.2^+^) and transplanted the cells into lethally irradiated B6.SJL recipient mice (CD45.1^+^) with host competitor BM cells (Figure 5a). The recipient mice were then monitored by CBC and CD45.2 chimerism was assessed. After 4 months, the transplanted mice showing comparable levels of CD45.2 chimerism were selected, irradiated with 450cGy TBI, and then monitored for CD45.2 chimerism (Figures 5a and b). During the regenerative phase, the CD45.2 chimerism in mice transplanted with WT KSL remained at levels comparable to pre-TBI. In contrast, we found a significant increase in CD45.2 chimerism in mice transplanted with Camkk2 null HSC (Figure 5c and Supplementary Figure S7). These findings provide direct evidence for a cell-intrinsic function of Camkk2 in regenerating HSPC because Camkk2 was ablated only in the transplanted KSL CD34- cells.

### CaMKK2 couples radiation signaling with AMPK anti-proliferative pathways

**Figure 6:**
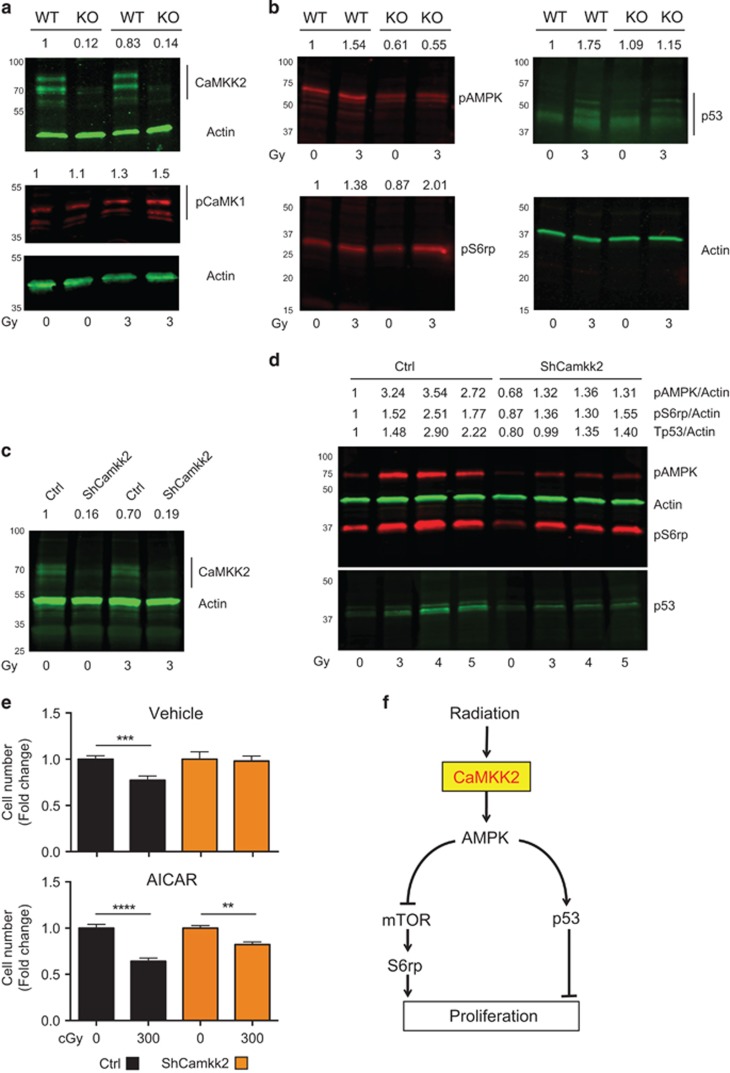
CaMKK2 couples radiation signaling with the AMPK anti-proliferative pathway. HSPC (KL+KSL) were isolated from WT and Camkk2 null (KO) mice and irradiated *in vitro* with 400cGy or left non-irradiated. Cells were then cultured for 1-h in regular medium. Protein expression was normalized by actin and expressed as fold change over basal (non-irradiated WT HSPC), and is reported on the top of each lane. (**a**) Immunoblots of CaMKK2, phospho-CaMK1 (pCaMK1) and actin. (**b**) Immunoblots of Tp53, phosphorylated AMPK and S6rp (pAMPK and pS6rp, respectively). (**c**-**e**) M1 myeloid progenitor cells were transduced with lentiviral vectors expressing a short hairpin sequence for silencing Camkk2 or a control sequence (ShCamkk2 and Ctrl, respectively). Ctrl and ShCamkk2 M1 cells were then irradiated or left non-irradiated. One-hour after irradiation, M1 cell protein expression was assessed by immunoblotting. (**c**) Expression of CaMKK2 and actin. (**d**) Immunoblots of pAMPK, pS6rp, actin and Tp53 of M1 cells irradiated with increasing doses of radiation or left non-irradiated. (**e**) M1 cells transduced with Ctrl and ShCamkk2 lentiviral vectors were 300cGy irradiated and cultured for 24 h in regular medium in the presence or absence of AICAR (100 *μ*M), a cell permeable AMPK agonist (Top and lower, respectively). Cell number was determined using a colorimetric assay, and the results are expressed as fold change of non-irradiated cells cultured in the absence of AICAR. Bars graph reports mean±S.E.M. The experiments included in this figures were replicated at least three times. (**f**) Modeling the radiation-induced CaMKK2-dependent signal pathway. **P<0.01,***P<0.005, ****P<0.001

To investigate CaMKK2 function in radiation-induced signaling, we sorted lin^-^ cKit^+^ cells (HSPC) from the BM of control and Camkk2 null mice and irradiated the cells *in vitro*. The HSPC were then incubated for 60 min in culture medium and CaMKK2 and its known targets were evaluated in total cell lysates by immunoblotting. As expected, null mice lacked any detectable CaMKK2 protein in sorted HSPC (Figure 6a). We found CaMKK2 loss did not impair the level of phospho-CaMKI in non-irradiated or irradiated HSPC (Figure 6a). There was no CaMKIV or phospho-CaMKIV detected in homeostatic or irradiated cells (data not shown). Interestingly, Camkk2 null non-irradiated HSPC had significantly less phospho-AMPK and failed to induce phospho-AMPK after radiation (Figure 6b). These results identify AMPK as the primary canonical target of CaMKK2 in HSPC and demonstrate CaMKK2 is required for coupling early radiation-induced signaling with AMPK activation.

AMPK is an evolutionarily conserved energy sensor that has an important role in cell proliferation, growth and survival.^28^ AMPK is also an important negative regulator of the Raptor-TSC-mTOR-S6K1 signaling pathway that controls S6 ribosomal protein (S6rp) to positively regulate cell proliferation. We measured the level of phospho-S6rp in non-irradiated and irradiated HSPC and found comparable levels of phospho-S6rp in non-irradiated WT and Camkk2 null HSPC. In contrast, we detected more phospho-S6rp in irradiated Camkk2 null HSPC compared with WT HSPC (1.3- *versus* 2.3-fold change, respectively; Figure 6b).

The p53 protein is an important effector of radiation signaling and its role in the control of apoptosis, quiescence and proliferation of HSPC is well documented.^29, 30^ Moreover, AMPK is a relevant upstream activator of the p53 pathway.^31, 32^ Therefore, we hypothesized the CaMKK2-AMPK axis is involved in radiation signaling and regulates p53 stabilization. We measured p53 in WT and Camkk2 null HSPC irradiated *in vitro* with 300cGy or left non-irradiated. The non-irradiated HSC from WT and Camkk2 null mice expressed comparable low levels of p53 (Figure 6b). However, WT HSPC accumulated more p53 compared with Camkk2 null HSPC in response to radiation (Figure 6b).

To further validate CaMKK2 function in radiation-induced signaling, we used lentiviral vectors to silence the expression of Camkk2 (ShCamkk2) in the M1 myeloid progenitor cell line (Figure 6c). Camkk2 deficiency induced similar changes in the gene expression profile of KSL and M1 cells (Supplementary Figures S8A and S8B). We measured the levels of phospho-AMPK, phospho-S6rp and p53 in control and ShCamkk2 M1 cells exposed to increasing radiation doses (300-500cGy) or left non-irradiated (Figure 6d). The radiation injury increased the phospho-AMPK and p53 levels in M1 control cells. In contrast, a minor increase in the phospho-AMPK and p53 levels was detected in ShCamkk2 M1 cells (Figure 6d). Radiation increased phospho-S6rp levels in both control and ShCamkk2 M1 irradiated cells, which suggests M1 cells may have additional CaMKK2-independent signals that control phosphorylation of S6rp after radiation.

To determine the functional effects of Camkk2 deficiency in M1 cells, we exposed control and ShCamkk2 M1 cells to 300cGy with or without a cell permeable AMPK agonist and then determined the number of live M1 cell 24 h after irradiation. We found ionizing radiation decreased the proliferation of M1 control cells but did not affect ShCamkk2 M1 cells (Figure 6e, top). The addition of the AMPK agonist 5-aminoimidazole-4-carboxamide 1-*β*-d-ribofuranoside, (AICAR), to the culture media reverted the refractory phenotype of ShCamkk2 M1 cells (Figure 6e, bottom). There was no increase in cell death observed following radiation or AICAR treatment (data not shown). Collectively, these findings corroborate our hypothesis and demonstrate CaMKK2 is part of the signal pathway that activates AMPK/p53 signaling and mediates the anti-proliferative effect of radiation damage.

### Pharmacologic inhibition of CaMKK2 enhances hematopoietic regeneration *in vivo*

**Figure 7:**
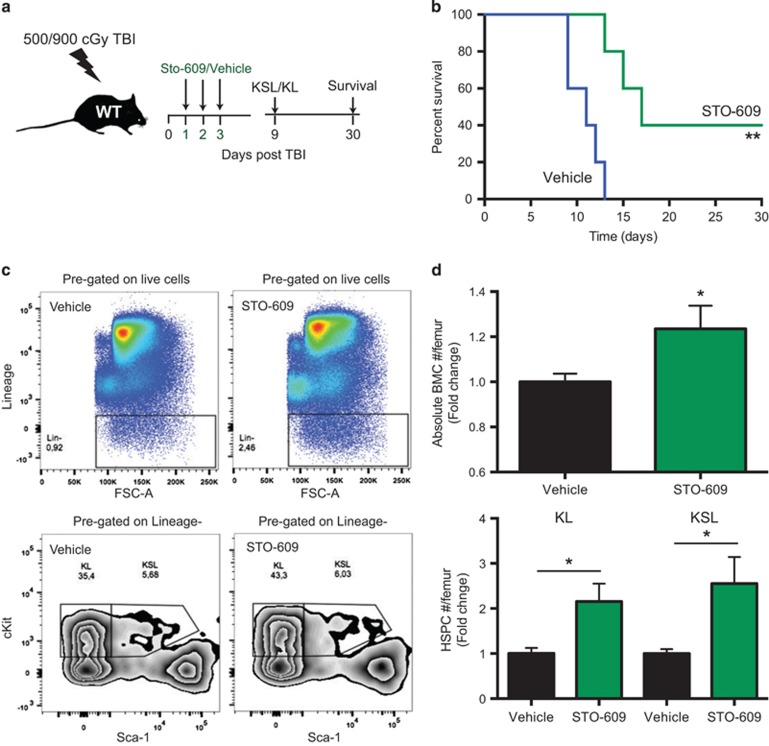
Pharmacologic inhibition of CaMKK2 enhances hematopoietic regeneration *in vivo*. Treatment with STO-609, a CaMKK2 inhibitor, mitigates the acute hematopoietic radiation syndrome. (**a**) Scheme of irradiation and STO-609 treatment in wild type mice. (**b**) Survival of 900cGy TBI wild-type mice treated with vehicle or STO-609 (*n*=10 per group). Wild-type mice were irradiated with 500cGy TBI and were then treated with STO-609 or vehicle after 24 h. Nine days after TBI, the mice were killed and bones were removed. The BM cells were counted and stained to identify HPSC (BMC). (**c**) Representative staining and gating strategy. (**d**) Absolute number of BMC and HSPC (upper and lower graph, respectively). The results are expressed as the fold changes over the absolute number of cells recovered form vehicle-treated wild type mice, and are normalized for one femur. Two independent experiments have been combined (total number of mice=9 per group). Bars graph reports mean±S.E.M. *P-value <0.05, **P-value <0.01

We hypothesized that pharmacologic inhibition of CaMKK2 increases survival and HSPC recovery following TBI. To test this relevant translational implication of our findings, we delivered a lethal radiation dose of 900cGy TBI (LD_100/15_) and then treated the animals with either vehicle or the selective CaMKK2 inhibitor STO-609. The administration of STO-609 at a dose of 10 *μ*M/kg on days 1, 2 and 3 post-TBI significantly prolonged survival in the LD_100/15_ model (Figures 7a and b). This dose of STO-609 was selected based on previous *in vivo* studies of the compound.^17, 33^ We next evaluated the effects of STO-609 treatment on HSPC of mice receiving 500cGy TBI and found more total BM cells and HSPC in the BM of mice 9 days after TBI (Figures 7c and d). Collectively, our findings indicate the transient administration of STO-609 after BM injury improves survival and expands HSPC.

## Discussion

Our results uncover an important role for CaMKK2 in the mechanism controlling HSPC regeneration. CaMKK2 controls transcriptomic programs associated with stem cell quiescence and its loss stimulates HSPC regeneration *in vivo*. Interestingly, we demonstrate that pharmacological inhibition of Camkk2 improves survival and accelerates HSPC recovery following hematopoietic radiation injury.

The interrelated processes of quiescence, proliferation and differentiation are tightly regulated in stem cells and this balance controls tissue regeneration after severe injuries.^26^ Extrinsic and HSC autonomous factors are involved in the fine tuning of these processes and defects in these molecular machineries are associated with high proliferative phenotype, significant decreases of HSC and progressive exhaustion of the hematopoietic compartment that culminates in premature death.^34, 35, 36^ We show Camkk2 deletion in HSPC significantly downregulates genes affiliated with the quiescent stem cell signature.^26, 27^ However, under homeostatic conditions Camkk2 null mice have only a slight decrease in HSPC number^19^ associated with mild alterations in blood cell counts. Camkk2 null mice do not develop a progressive exhaustion of the hematopoietic compartment or blood cancer with age (LR personal communication). These findings indicate Camkk2 loss does not affect HSPC under homeostatic conditions. On the contrary, Camkk2 null HSPC have a hyper-proliferative phenotype *in vitro* and an enhanced regenerative capability following BM damage. Interestingly, in conditions that mimic the functional cross talk in the niche, Camkk2 null KSL show higher functionality than WT cells. Collectively, these findings suggest CaMKK2 has a novel function in the signaling network involved in quiescence and the regenerative response of HSC in the niche.

We demonstrated CaMKK2 links proximal radiation signaling to activation of the anti-proliferative AMPK/p53 signaling pathways (Figure 6f). Under homeostatic conditions, CaMKK2 loss decreases phospho-T172 AMPK levels. This result suggests other upstream kinases such as Lkb1 contribute to AMPK regulation in quiescent HSC.^36, 37, 38^ The CaMKK2-AMPK axis is dispensable for maintaining homeostatic HSC, which is supported by the findings that germ-line deletion of Camkk2^19^ or deletion of Ampk in the hematopoietic compartment^38^ does not impair hematopoiesis. Conversely, Lkb1 deletion is associated with a transient hyper-proliferative HSC response that is followed by an AMPK/mTOR independent catastrophic HSC depletion, pancytopenia and animal death.^36, 37, 38^ Ionizing radiation regulates AMPK activation in endothelial cells,^39^ mouse embryonic fibroblasts (MEFs)^40^ and cancer cells.^41, 42^ However, the effect of radiation on AMPK in HSPC is poorly understood and the predominant upstream kinase responsible for radiation-induced AMPK activation is unknown. Our results show CamKK2 may be the critical upstream kinase and we demonstrate radiation activates AMPK in HSPC via CaMKK2. Camkk2 deletion prevents the inhibitory effects exerted by acute activation of AMPK on downstream effectors of mTOR signaling^43^ and p53,^31, 32^ which control HSC proliferation under stress.^19, 36, 37, 38, 43, 44, 45^ The protein p53 is an important effector of the anti-proliferative effects of radiation and studies have shown short-term p53 inhibition following radiation damage facilitates hematopoietic recovery and prevents development of radiation-induced lymphomas.^46, 47, 48^ Importantly, p53 is a target of AMPK kinase. Here, we demonstrate for the first time that CaMKK2 is required for p53 accumulation following radiation injury.

Our findings have significant translational impact and suggest pharmacological CaMKK2 inhibition accelerates hematopoietic recovery following TBI injury. Furthermore, CaMKK2 inhibition may prevent blood cancers associated with the radiation treatment. Pharmacological p53 inhibition has been proposed to attenuate acute toxicity and decrease the risk of radiation-related blood cancer development.^46^ However, p53 is ubiquitously expressed which may limit therapeutic approaches. Conversely, CaMKK2 is restricted to a limited number of cell types including HSPC and myeloid cells. The restricted cellular expression of CAMKK2 makes it an attractive target to selectively prevent radiation-induced upregulation of p53 in HSPC and could limit the effects of p53 activation while simultaneously reducing the potential side effects associated with systemic p53 inhibition.

## Materials and methods

### Mice

CaMKK2-EGFP transgenic reporter mice have been described previously.^49^ Radiation was delivered by a Shepherd Cs^137^*γ*-irradiator at a dose rate of approximately 600cGy per min. All animal experiments were performed according to protocols approved by the Duke University Institutional Animal Care and Use Committee.

### Antibodies

Anti-CaMKK (pan-KK) was from BD Biosciences (San Jose, CA, USA), anti-phospho-S6rp^S240/244^, anti-phospho-AMPK*α*^T172^ and anti-AMPK*α* were from Cell Signaling Technology (Danvers, MA, USA). Anti-phospho-CaMKI^T177^ was from Santa Cruz Biotechnology (Dallas, TX, USA). Anti-*β*-actin was from Sigma.

### M1 cells and proliferation assay

M1 cells (ATCC TIB-192) were obtained from Duke Cell Culture Facility (Durham, NC, USA). In proliferation experiments, M1 cells were seeded at 2 × 10^4^/ml per well into a 24-well plate. MTS Cell Proliferation Assay Kit was from Abcam (Cambridge, MA, USA).

### Isolation and FACS of HSPC

HSPC were sorted from mouse BM based on surface expression of c-Kit, Sca-1, and low to negative expression of lineage markers (lin^-^). KSL CD34^-^ were used in reconstitution experiment as described.^50^ All antibodies were purchased from BD Biosciences, BioLegend (San Diego, CA, USA) or eBioscience (Waltham, MA, USA). Annexin-V/7AAD apoptosis kit was from BD Biosciences. The cell sorting and analyses were performed on a FACSVantage cell sorter or CANTO analyzer (Becton Dickinson, Franklin Lakes, NJ, USA).

### Microarray analysis

KLS cells were sorted from BM directly into Buffer RLT (Qiagen, Frederick, MD, USA). Microarray analysis was performed at Sequencing and Genome Technologies Shared Resource (Duke University). Microarray data are available at GEO (accession number: GSE95733).

### Gene set enrichment analysis

GSEA^22^ was applied to our microarray data. All genes were ranked by the fold change between the Camkk2 null and WT control samples. Normalized enrichment score (NES) and adjusted q-values were computed utilizing the GSEA method, based on 1000 random permutations of the ranked genes. Gene set collections (MSigDB, http://www.broadinstitute.org/gsea/msigdb/) was used to determine enriched pathways.

### *In vivo* transplantation experiments

For competitive engraftment assays, the total BM cells were isolated from femurs and tibiae of WT and Camkk2 null (CD45.2 background). c-Kit-positive cells were enriched using anti-CD117/c-Kit microbeads (Miltenyi Biotec, Auburn, CA, USA). The KSL CD34^-^ stem cells (CD45.2) were then sorted and 1000 cells were injected with 5x10^5^ competitor whole BM (CD45.1) into lethally irradiated (split dose totaling 10Gy) recipient B6-CD45.1 (B6.SJL-Ptprca Pepcb/BoyJ) mice via the retro-orbital sinus. In the same experiments, KSL CD34^-^ cells were sorted from BM of Camkk2 null or control mice treated with 200cGy 90 days before transplant. For lineage analysis, peripheral blood cells were collected and prepared as described.^51^ In some experiments, transplanted mice were monitored for up to 4 months, and then mice received 450cGy TBI. Mice were finally monitored for chimerism.

### *In vitro* proliferation with endothelial cells and methylcellulose assays

Primary BMECs were generated as described.^8^ For liquid culture experiments, freshly sorted KSL cells from control or Camkk2 null mice were plated in 2% FCS-medium (X-Vivo15, Lonza, Portsmouth, NH, USA) supplemented with 50 *μ*M 2-mercaptoethanol, SCF (50 ng/ml) and Flt-3 (30 ng/ml). After culturing for the indicated time, live cells were counted using Trypan blue exclusion. For methylcellulose assays, the recovered cells were plated in complete methylcellulose medium (StemCell Technologies, Vancouver, BC, Canada, catalog number M3434). The colony numbers were counted 8–10 days after plating.

### Immunofluorescence staining of bone sections

Femurs were decalcified, embedded in OCT media (Sakura Finetek, Torrance, CA, USA) and 10 *μ*m sections were cut using the CryoJane tape system (Instrumedics Inc., Hackensack, NJ, USA) as described.^52^ To assess BM cellularity, sections were stained with hematoxylin/eosin. Vasculature and EGFP+ cells were identified with anti-mouse VE-Cadherin (Abcam) and anti-GFP antibodies (Abcam). The nuclear dye DAPI (Invitrogen, Waltham, MA, USA) was included in all stains. Images were obtained using an Axiovert 200 microscope (Carl Zeiss, Thornwood, NY, USA).

### Immunoblotting

Immunoblots were performed according with the protocol described previously,^49^ and visualized on an Odyssay CLx imager (LI-COR Biosciences, Lincoln, NE, USA). Image Studio software (Lincoln, NE, USA) was used for quantitation.

### ShRNA lentivirus

shRNA3145 (TRCN0000028776) against Camkk2 and the negative control pLKO (TRCN0000241923) were obtained from Open Biosystems (Huntsville, USA). M1 cells were infected with shRNA3145 or pLKO virus containing sham shRNA pLKO vector, or *Camkk2* (3142, 3143 or 3145 clones) shRNA vector using polybrene as per the manufacturer’s instruction. Cells were then selected and maintained in media with puromycin.

### Statistical analysis

In mice studies, a power calculation of sample size was performed with the help of the Duke University Biostatistic core service. A two-tailed Student’s *t*-tests and one-way ANOVA were used to determine statistical significance. A *P*-value <0.05 was considered significant. The variance was comparable between each group of data that was statistically compared. The statistical significance was determined using GraphPad Prism 7 software (GraphPad Software, Inc., La Jolla, CA, USA).
