# Supplementary material for: Calcium/calmodulin-dependent kinase kinase 2 regulates hematopoietic stem and progenitor cell regeneration
Source: Cell Death Dis. 2017 Oct 5;8(10):e3076–. doi: 10.1038/cddis.2017.474 (PMC5680595; doi:10.1038/cddis.2017.474)
Supplement: Supplementary Information [file cddis2017474x1.pdf]

Supplemental materials

Calcium/Calmodulin Dependent Kinase Kinase 2 (CaMKK2) regulates hematopoietic stem and progenitor cell regeneration

Luigi Racioppi[1,2^*], William Lento[^1], Wei Huang[1], Stephanie Arvai[1], Phuong L. Doan[1], Jeffrey R Harris[1], Fernando Marcon[3], Helder I Nakaya[3], Yaping Liu[1], Nelson Chao[1].

Supplemental methods

Pathway analysis

iPathwayGuide® (IPG) software use the impact analysis method with Bonferroni correction. Briefly, this method uses two types of evidence: i) the over-representation of differentially expressed (DE) genes in a given pathway and ii) the perturbation of that pathway computed by propagating the measured expression changes across the pathway topology. These aspects are captured by two independent probability values, pORA and pAcc, that are then combined in a unique pathway-specific p-value. The underlying pathway topologies, comprised of genes and their directional interactions, are obtained from the KEGG database.

RNA isolation and Real-Time PCR

Total RNAs were isolated using QIAquick PCR purification kit (Qiagen, Valencia, CA). Single-stranded cDNA was synthesized using SuperScript II reverse transcriptase (Invitrogen) according to the manufacturer's directions and protocol described previously[1]. Real-time PCRs were performed using an iCycler (Bio-Rad) with the IQ SYBR Green supermix (Bio-Rad). After deriving the relative amount of each transcript from a standard curve, transcript levels were normalized to GAPDH. PCR primers were from Qiagen (RT^2 quantitative PCR primer assays, SAbiosciences).

Supplemental Table 1

Top Genes Upregulated in Camkk2 null KSL

Gene symbol	logfc	adjpv
Cldn13	-4.59683555	0.000001
Ahsp	-4.53838915	0.000001
Rhag	-4.40659729	0.00001
S100a8	-4.31267656	0.000187
Aqp1	-3.59649001	0.000004
Ces2g	-3.54673969	0.000001
Ermap	-3.40552135	0.000001
Gm5843	-3.38201456	0.000001
Slc38a5	-3.28166281	0.000001
Klf1	-3.27487802	0.000007
Rhd	-3.27146828	0.000005
Nxpe2	-3.26499576	0.000001
Tspan8	-3.10727365	0.000003
Gm15915	-3.08053288	0.000012
Trem3	-3.06432507	0.000005
Fam132a	-3.02237421	0.000002
Car1	-3.01798288	0.000001
Ms4a3	-3.00716665	0.000004
Ly6c2	-2.90853906	0.000036
Slc25a21	-2.86505395	0.000001
Atp1b2	-2.8255369	0.000004
Tspan33	-2.62249231	0.000001
Epor	-2.60490944	0.000002
Cpox	-2.5525653	0.000001
Mt1	-2.51608227	0.000004
Ccne1	-2.50143595	0.000009
Snora73b	-2.49650579	0.000067
Ctse	-2.40964417	0.011323
Elane	-2.403335	0.000012
Paqr9	-2.403335	0.000048
Kel	-2.38508632	0.000001
Snora73a	-2.33020026	0.000047
Hmbs	-2.30657711	0.000001
Asns	-2.29757255	0.000001
Ppap2a	-2.10900034	0.000001
Gypa	-2.07317193	0.000009
Spire1	-2.02946317	0.000002

Igsf6	-2.02814825	0.000168
Stom	-2.0255132	0.000038

Top Genes Downregulated in Camkk2 null KSL

Ighv1-2	7.15251158	0.000076
Igkv19-93	6.08258414	0.003751
Dntt	4.83429581	0.000001
Ighm	4.54881145	0.000004
Gm19590	4.53270701	0.000001
Eltd1	3.88341775	0.000006
Gcnt2	3.74525979	0.000004
Igh-VJ558	3.65970808	0.001526
Ctla2b	3.60604118	0.00001
Gm5111	3.57962247	0.000001
Igkv4-59	3.50735758	0.000208
Ighv1-77	3.45852245	0.000417
Insl6	3.41015712	0.000006
Flt3	3.40585319	0.000003
Ctla2a	3.28053521	0.000018
Ighv11-1	3.24454357	0.000014
Igkv4-54	3.15316328	0.00007
Igkv4-62	3.15230858	0.000019
Myct1	3.07176696	0.000001
Igkv8-30	2.98061864	0.033687
Gpr56	2.97654945	0.000008
Igj	2.95543098	0.000546
Igkv4-57	2.93916192	0.000018
Laptm4b	2.92477319	0.000152
Meis1	2.8384935	0.000001
Ighv1-73	2.83203618	0.000539
Ighv1-55	2.83085763	0.000978
Igkv4-55	2.77757626	0.000211
Cd34	2.74019465	0.000002
Igkv4-61	2.7304638	0.000258
Rbp1	2.69665216	0.000001
Ighv1-5	2.69056489	0.0049
H2-Ob	2.67759099	0.000001
Ifi44	2.63905733	0.003045
Sox4	2.61812549	0.000001
Tmem176b	2.61666564	0.000001

9030619P08Rik	2.60786147	0.000005
Igkv4-57-1	2.60638655	0.000214
Angpt1	2.60564827	0.000001

Supplemental Tab 2

Pathway analysis

pName	pv_bonferroni
Lysosome	7.92E-10
Hematopoietic cell lineage	7.73E-08
Cell adhesion molecules (CAMs)	8.22E-08
Metabolic pathways	5.73E-07
Phagosome	2.08E-05
Natural killer cell mediated cytotoxicity	6.41E-05
Fc gamma R-mediated phagocytosis	0.000116349
Antigen processing and presentation	0.000133403
Pathways in cancer	0.000158135
Tuberculosis	0.000437487
Fc epsilon RI signaling pathway	0.000504321
Leishmaniasis	0.000580196
Cytokine-cytokine receptor interaction	0.000777057
Transcriptional misregulation in cancer	0.000792215
Staphylococcus aureus infection	0.00098973
FoxO signaling pathway	0.001262219
Viral myocarditis	0.001352085
Toxoplasmosis	0.001699385
Inflammatory bowel disease (IBD)	0.002013243
Influenza A	0.002205047
Hepatitis B	0.002251295
Herpes simplex infection	0.002351594
Apoptosis	0.002375065
HTLV-I infection	0.00267446
Glutathione metabolism	0.002954605
Type I diabetes mellitus	0.003012121
Rheumatoid arthritis	0.003049457
Graft-versus-host disease	0.003508518
B cell receptor signaling pathway	0.003607519
Allograft rejection	0.003775925
T cell receptor signaling pathway	0.003837721
Jak-STAT signaling pathway	0.00404303
Osteoclast differentiation	0.004059177
Leukocyte transendothelial migration	0.005472502
Chagas disease (American trypanosomiasis)	0.006290182
Sphingolipid signaling pathway	0.008017898

Viral carcinogenesis	0.011033526
Measles	0.012717421
Central carbon metabolism in cancer	0.013339102
Ras signaling pathway	0.017951374
Systemic lupus erythematosus	0.020248129
ABC transporters	0.021274373
MAPK signaling pathway	0.021903743
Chemokine signaling pathway	0.027561406
Intestinal immune network for IgA production	0.036701637

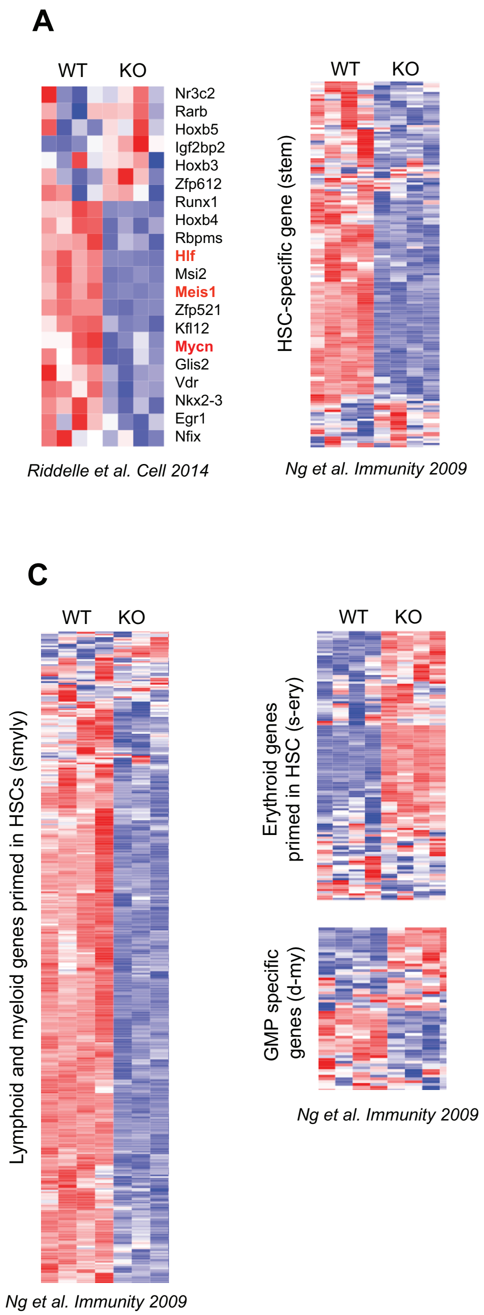

Supplemental Figure S1. Camkk2 differentially modulate stem cell- and lineage-associated transcriptional programs. Heatmap representation showing differentially expressed genes in: (A) hematopoietic stem cells; (B) immediate downstream progenitors; (C) lineage-affiliated genes in Camkk2 KO compared to WT KSL. Genes in bold red text are reported to induce reprogramming of differentiated hematopoietic cells into induced HSCs. The color key for all heatmaps indicates row-wise scaled RPKM values (z-score).

A

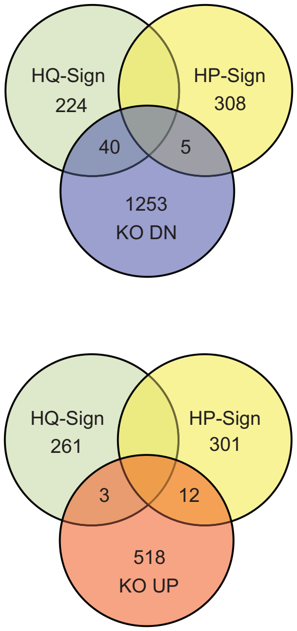

B

Signature	Non change[1]	KO DN[2]
HQ-Sign	224	40
HP-Sign	308	5

1, Non significant change in KO compared to WT;
2, Down regualted in KO compared to WT;
P = 0.0001

Signature	Non change[1]	KO UP[2]
HQ-Sign	261	3
HP-Sign	301	12

1, Non significant change in KO compared to WT;
2, Up regulared in KO compared to WT;
P = 0.0632

C

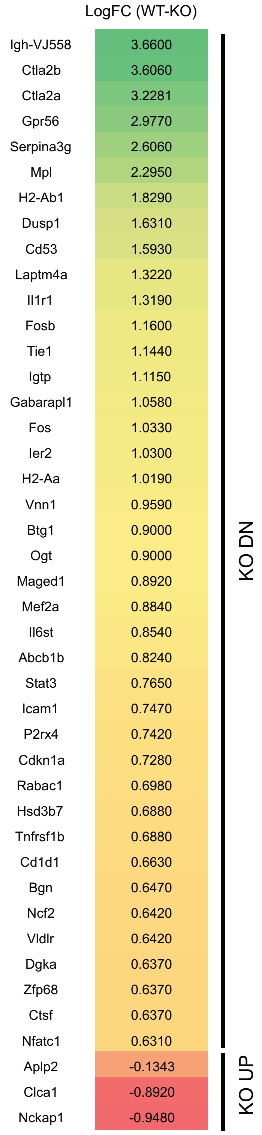

Venezia et al.Plos Biology 2004

Supplemental Figure S2. Genes affiliated with hematopoietic stem cell quiescence are down regulated in Camkk2 null KSL. (A) Venn diagram showing the overleaping of genes down or up regulated in Camkk2 null KSL compared to WT (KO DN and KO UP, respectively) with genes affiliated with quiescent or proliferating hematopoietic stem cells (HQ-Sign and HP-Sign, respectively). (B) Genes affiliated with hematopoietic stem cell quiescent signature are significantly downregulated in Camkk2 null KSL (p = 0,0001). (C) Loss of Camkk2 downregulates the genetic quiescent signature in stem cells. Fold chance of HQ-Sign genes significantly downregulated in Camkk2 null KSL compared to WT genes. Venny 2.0[2] was used for Venn diagram analyses.

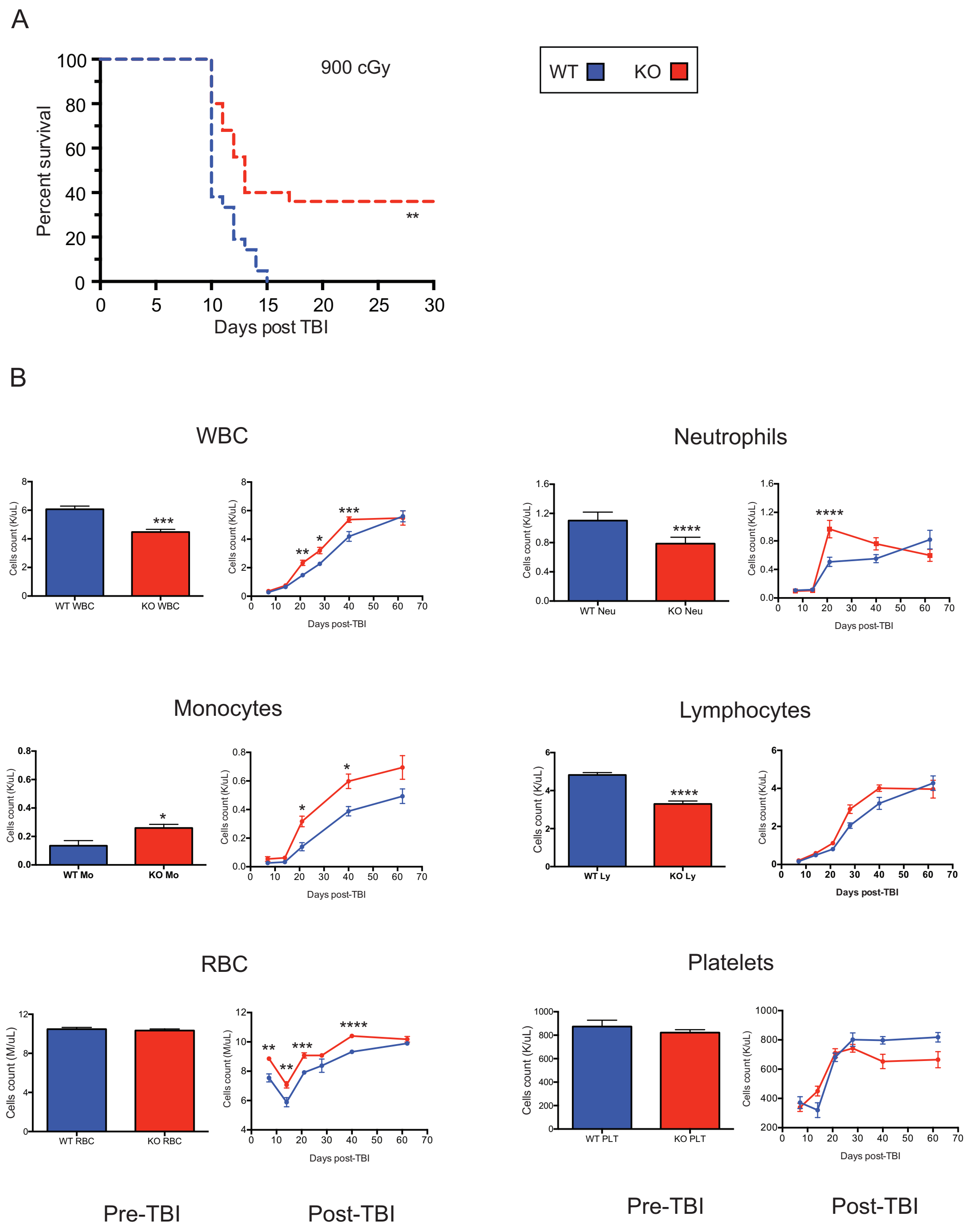

Supplemental Figure S3. Camkk2 null mice have improved survival and accelerated hematopoietic recovery following total body irradiation. Mice were TBI and monitored for survival and blood cell count recovery. (A) Survival of WT and Camkk2 null mice (WT and KO, respectively) irradiated with 800cGy (n = 13 mice/genotype). Cell blood count number in WT and KO mice TBI with 700 cGy and monitored by CBC (n = 6 and 9 for WT and KO group, respectively). The absolute numbers +/- SEM are shown. Bars graphs show pre-TBI CBC. *p<0.05, **p<0.01, ***p<0.005. **** p<0.001.

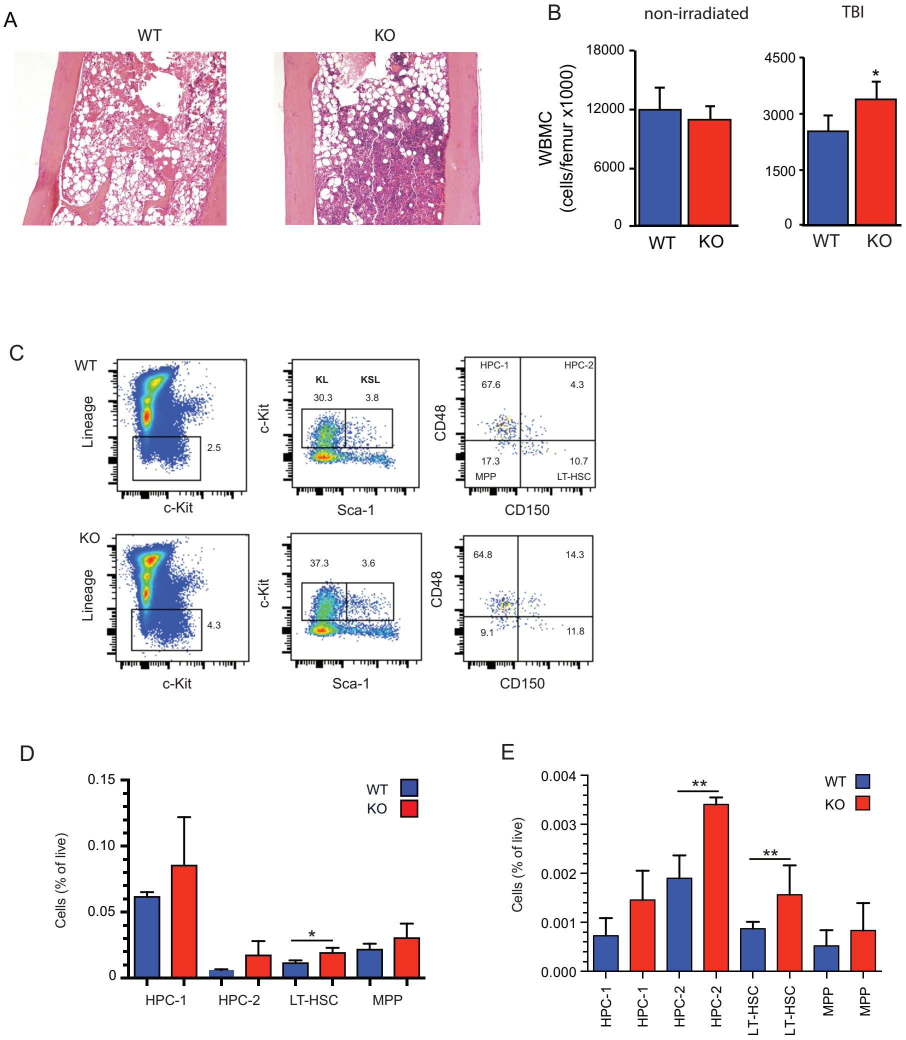

Supplemental Figure S4. Camkk2 null mice have an accelerated bone marrow recovery following total body irradiation. WT and KO mice were irradiated with 700cGy TBI and sacrificed on day 14. (A) Representative hematoxylin and eosin staining of WT and Camkk2 null mice (WT and KO, respectively) femur sections. (B) Absolute count number of white BM cells (WBMC) isolated from femurs of non-irradiated and TBI mice (left and right bar graphs, respectively; n = 10 mice/group). Data refers to cells recovered from one bone. (C) Gating strategy to identify SLAM KSL cells. Percentage of SLAM KSL in non-irradiated and TBI WT and KO mice (D and E panels, respectively; n = 6 mice/genotype). Bars graph reports mean +/- SEM. * p-values < 0.05; ** p-values <0.01.

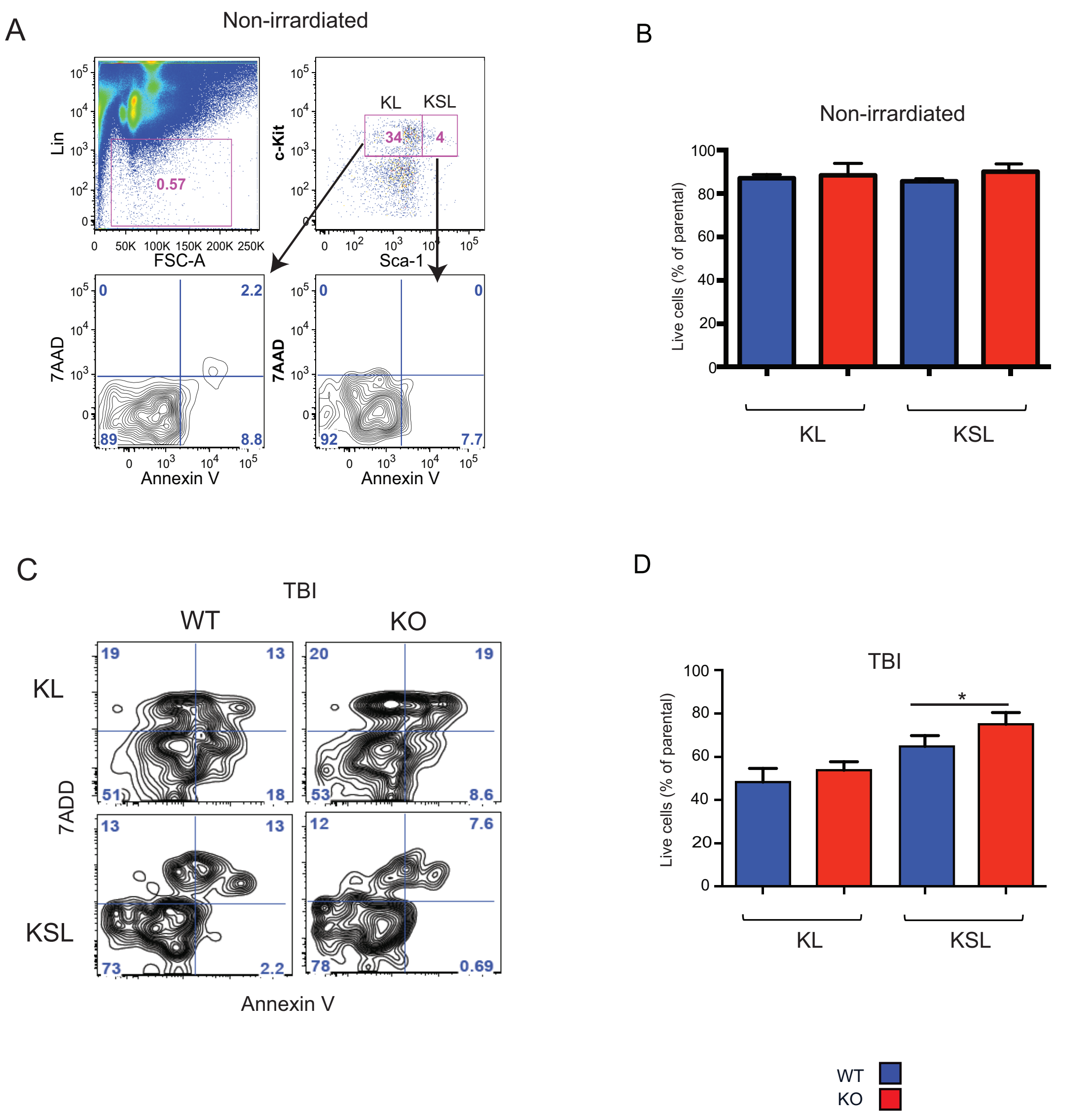

Supplemental Figure S5. Effect of genetic ablation of Camkk2 on HSPC survival under homeostatic conditions and following total body irradiation. (A) gating strategy to identify KL and KSL live and death cells by using Annexin-V and 7AAD in bone marrow of non-irradiated WT and Camkk2 null mice (WT and KO, respectively). (B) Percentages of live KSL and KL cells in non-irradiated WT and Camkk2 null mice (n = 6 mice/genotype). (C) WT and KO mice were euthanized 24-hours after 450cGy TBI and apoptotic and live KL and KSL cells were identified by flow cytometry. (D) The percentage of Annexin V-/7AAD- live cells in irradiated WT and KO mice is shown (n = 6/genotype). Bars graph reports mean +/- SEM. * p-values < 0.05.

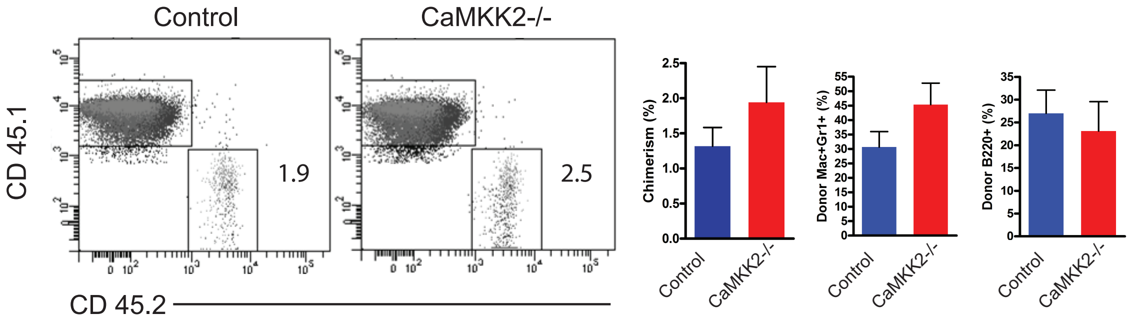

Supplemental Figure S6. Loss of Camkk2 does not impair the engraftment potential of irradiated hematopoietic stem cells. Control and Camkk2 null mice received 200cGy TBI and were allowed to regenerate. KSLCD34$^-$ cells were then sorted from the irradiated donors and transplanted into lethally irradiated recipient mice with competitor bone marrow. The recipient mice were bled at 8 weeks and donor CD45.2 chimerism was analyzed by flow cytometry. There was no significant difference in peripheral blood chimerism at 8 weeks. The data indicate the accelerated regeneration found in irradiated Camkk2 null HSCs is not associated with decreased transplantation function or malignant transformation. Bars graph reports mean +/- SD; n = 6 mice/group).

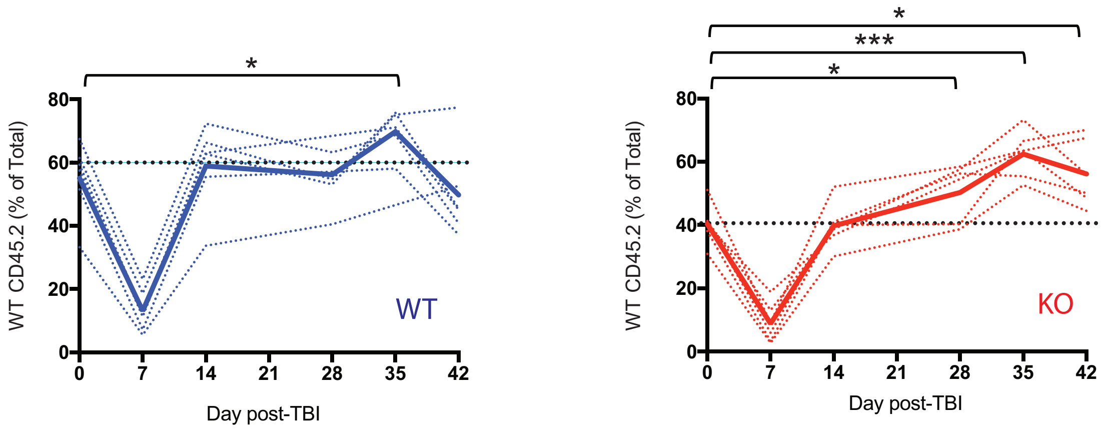

Supplemental Figure S7. Camkk2 null HSCs have a cell-intrinsic enhanced regenerative capability *in vivo*. KSLCD34⁻ cells were isolated from WT and KO and transplanted in lethally irradiated recipient mice with CD45.1 competitor bone marrow. The recipient mice receiving WT or KO KSLCD34⁻ cells were monitored for 4 months. Subsequently, mice showing comparable percentages of WT or KO donor's CD45.2 cells were irradiated with 450cGy TBI and bled weekly after irradiation. Donor CD45.2 chimerism was monitored by flow cytometry and the results expressed as fold change over the basal level (pre-TBI). (A) Scheme of the experiment. (B) CD45.2 chimerism in mice reconstituted with WT or KO KSLCD34⁻ before TBI. (C) Kinetics of CD45.2 chimerism. Blue and red dotted lines indicate CD45.2 chimerism in individual mice transplanted with WT or KO KSLCD34⁻ cells, respectively. Blue and red bold lines indicate the average of CD45.2 fold change in WT and KO group, respectively (n= 6 mice /group). Bars graph reports mean +/- SEM. * p<0.05, ** p<0.01, *** p<0.05.

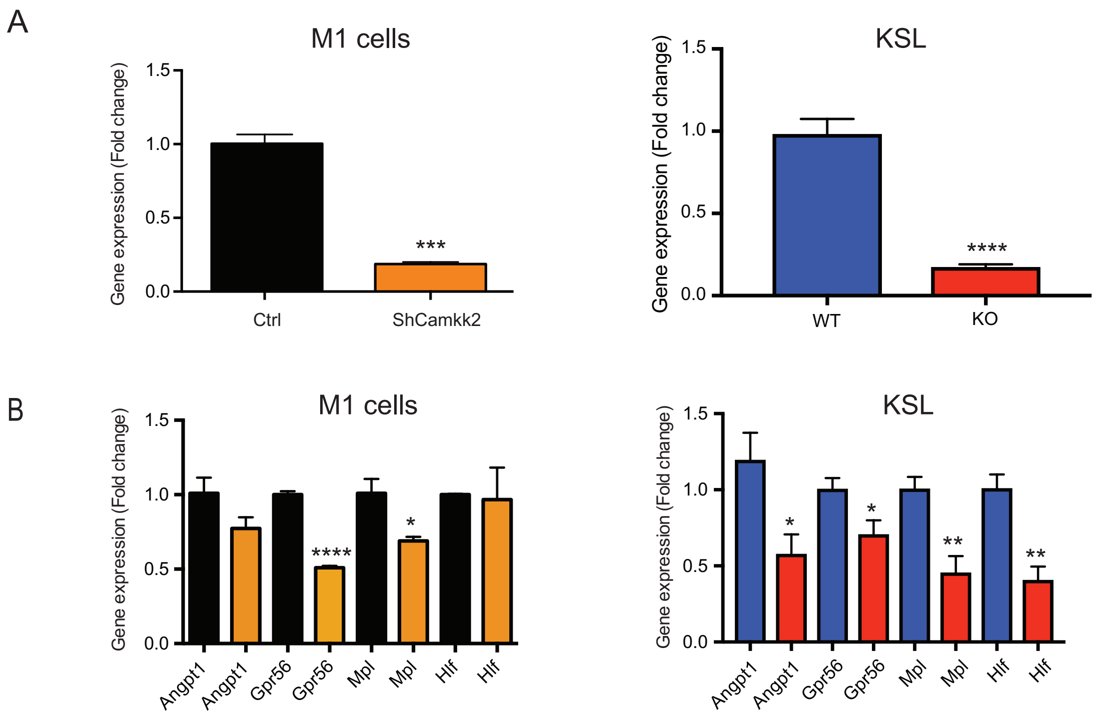

Supplemental Figure S8. Modeling CaMKK2 deficiency in the myeloblastic M1 cell line. M1 cells were transduced with a lentiviral vector or expressing a control sequence or a short hairpin sequence for silencing Camkk2 (Ctrl and ShCamkk2, respectively). (A) Normalized Camkk2 mRNA expression in M1 cells transduced with control or ShCamkk2 vectors (left; n = 6). Camkk2 gene expression in fresh isolated WT and Camkk2 null (KO) KSL (right; n = 9). (B) Normalized gene expression in transduced M1 cells and primary WT and KO KSL. The selected genes were highly expressed in both cell types (M1 and KSL), and differentially expressed (DEGs) KO KSL compared to WT. Silencing of Camkk2 in M1 cells and Genetic ablation of CaMKK2 has similar effects on gene expression. Bars graph reports mean +/- SEM. * p<0.05, ** p<0.01, *** p<0.05, **** p<0.05.

References

1 Racioppi, L., Noeldner, P. K., Lin, F., Arvai, S. & Means, A. R. Calcium/calmodulin-dependent protein kinase kinase 2 regulates macrophage-mediated inflammatory responses. *J Biol Chem* **287**, 11579-11591, doi:10.1074/jbc.M111.336032 (2012).

2 *Oliveros, J. C. Venny. An interactive tool for comparing lists with Venn's diagrams.*, <http://bioinfogp.cnb.csic.es/tools/venny/index.html> (*2007-2015*).
